# Supplementary material for: Associations between Four Diet Quality Indexes and High Blood Pressure among Adults: Results from the 2015 Health Survey of Sao Paulo
Source: Nutrients. 2024 Feb 24;16(5):629. doi: 10.3390/nu16050629 (PMC10935342; doi:10.3390/nu16050629)
Supplement: Supplementary file 1 [file nutrients-16-00629-s001.zip › nutrients-2863310-supplementary.pdf]

Figure S1. Sample flowchart in the 2015 Health Survey of São Paulo with Focus on Nutrition (2015 ISA-Nutrition) eligible for the present study.

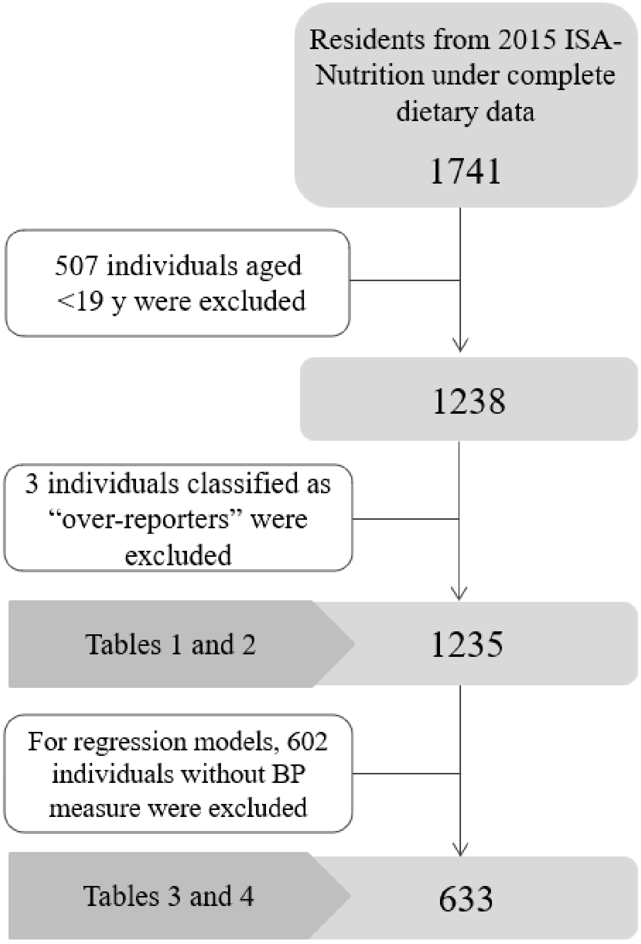

Table S1. Components and scoring of the AHEI, HEI-2020, BHEI, and DASH diet quality scores.

	**AHEI[1]**		**HEI-2020[1]**		**BHEI[1]**		**DASH**	
	Standard for minimum score	Standard for maximum score	Standard for minimum score	Standard for maximum score	Standard for minimum score	Standard for maximum score	Standard for minimum score	Standard for maximum score
**Adequacy Components**								
Total fruit	–	–	0	≥0.8 cups equivalents /1,000 kcal	0	≥1 serving /1,000 kcal	Quintile 1	Quintile 5
Whole fruit	0	≥4 servings/d	0	≥0.4 cups equivalents /1,000 kcal	0	≥0.5 serving /1,000 kcal	–	–
Total vegetables	0	≥5 servings/d	0	≥1.1 cups equivalents /1,000 kcal	0	≥1.0 serving /1,000 kcal	Quintile 1	Quintile 5
Greens and Beans	–	–	0	≥0.2 cups equivalents /1,000 kcal	–	–	–	–
Dark green and orange vegetables and legumes	–	–	–	–	0	≥0.5 serving /1,000 kcal	–	–
Total grains	–	–	–	–	0	≥2 servings /1,000 kcal	–	–
Whole grains	0	≥90 g/d (m) ≥75 g/d (w)	0	≥1.5 oz equivalents /1,000 kcal	0	≥1 serving /1,000 kcal	Quintile 1	Quintile 5
Total dairy products	–	–	0	≥1.3 cups equivalents /1,000 kcal		≥1.5 servings /1,000 kcal	–	–
Low-fat dairy	–	–	–	–	–	–	Quintile 1	Quintile 5
Nuts, seeds, legumes	0	≥1 serving/d	–	–	–	–	Quintile 1	Quintile 5
Total protein foods	–	–	0	≥2.5 oz equivalents /1,000 kcal	0	≥1 serving /1,000 kcal	–	–
Seafood and plant proteins	–	–	0	≥0.8 oz equivalents /1,000 kcal	–	–	–	–
Polyunsaturated fatty acids	≤2 % energy/d	≥10 % energy/d	(PUFAs+MUFAs) / SFAs≤1.2	(PUFAs+MUFAs) / SFAs≥2.5	–	–	–	–
Oils	–	–	–	–	0	≥0.5 serving /1,000 kcal	–	–
Long-chain n-3 fats	0 mg/d	≥250 mg/d	–	–	–	–	–	–
**Moderation Components**								
Refined grains	–	–	≥4.3 oz equivalents /1,000 kcal	≤1.8 oz equivalents /1,000 kcal	–	–	–	–
Red and processed meats	≥1.5 serving/d	0 servings/d	–	–	–	–	Quintile 5	Quintile 1
Sugar-sweetened beverages	≥1 serving/d	0 servings/d	–	–	–	–	Quintile 5	Quintile 1

Added sugar	–	–	≥26% of energy	≤6.5% of energy	–	–	–	–
Alcoholic beverages[2]	≥3.5 drinks/d (m) ≥2.5 drinks/d (w)	0.5-2.0 drinks/d (m) 0.5-1.5 drinks/d (w)	–	–	–	–	–	–
Saturated fatty acids	–	–	≥16% of energy	≤8% of energy	≥15% of energy	≤7% of energy	–	–
Trans fat	≥4 % energy/d	≤0.5 % energy/d	–	–	–	–	–	–
Calories from solid fats, alcoholic beverages, and added sugars	–	–	–	–	≥35% of energy	≤10% of energy	–	–
Sodium	Highest decile	Lowest decile	≥2g /1,000 kcal	≤1.1g /1,000 kcal	≥2g /1,000 kcal	≤0.7g /1,000 kcal	Quintile 5	Quintile 1
**Range of scores**	0	110	0	100	0	100	8	40

Abbreviations: MUFA, monounsaturated fatty acid; PUFA, polyunsaturated fatty acid; SFA, saturated fatty acid; m, men; w, women.

[1] Intakes between the minimum and maximum levels are scored proportionately.

[2] In the design of the AHEI, authors assigned the highest score to moderate, and the lowest score to heavy, alcohol consumers. The nondrinkers received a score of 2.5. One drink is 4 oz of wine, 12 oz of beer, or 1.5 oz of liquor (1 oz = 28.35 g).
